# Efficacy and effectiveness of 20 child health interventions in China: Systematic review of Chinese literature

**Published:** 2011-06

**Authors:** Jian Shayne F. Zhang

**Affiliations:** Centre for Population Health Sciences and Global Health Academy, University of Edinburgh, Scotland, UK

## Abstract

**Aim:**

The research production of the Chinese academics for the past few decades, which is being published in more than nine thousands of Chinese academic periodicals, has recently been digitalized and made available in the public domain. The aim of this study was to systematically identify and assess the evidence from Chinese literature sources on the efficacy and effectiveness of child health interventions in China.

**Methods:**

The Chinese National Knowledge Infrastructure databases were searched for the studies with primary data on efficacy or effectiveness of child health interventions in China between 1980 and 2011. The searches of PubMed and the ‘Lives Saved Tool (LiST)’ evidence base were also performed to identify the counterpart evidence in the English language.

**Results:**

Of 32 interventions initially identified in the Chinese literature, 20 interventions sustained the primary information addressing efficacy or effectiveness. Among preventive interventions (14 interventions), most studies were dedicated to complementary feeding (7 studies), kangaroo mother care (7 studies) and syphilis detection and treatment (4 studies). Among treatment interventions (6 interventions), the most frequently studied were zinc for treatment of diarrhoea (11 studies) and newborn resuscitation (9 studies). The evidence on efficacy or effectiveness of the 32 interventions conducted in Chinese children in the Chinese literature was either of comparable quality, or more informative than the available reports on China in the English literature, which rarely contained studies on child health intervention effectiveness exclusively in Chinese population. The included studies reported positive results unanimously, implying a likely publication bias.

**Discussion:**

The evidence on the efficacy and effectiveness of child health interventions in China is typically modest in quantity and quality, and implies a notable urban-rural discrepancy in applied health systems research to improve child health interventions and programmes. However, it is clear that considerable research interests and initiatives from both inside and outside the country have been concentrating on implementation, long-term operation, evaluation and further development of child health interventions, especially preventive interventions in China.

In 2000, the UN defined a set of goals on which a global political consensus was reached – the ‘Millennium Development Goals’ (1,2). The fourth goal was defined as an ambition to decrease the levels of global child mortality between 1990 and 2015 by two thirds (3). The leading strategies for achieving child mortality reduction were to implement cost-effective interventions in the largest part of the population in low and middle income countries (4-7). These interventions were developed to prevent and treat the leading causes of child mortality, eg, preterm birth complications, birth asphyxia, neonatal infections, pneumonia, congenital abnormalities, diarrhoea, tetanus and HIV/AIDS (5,6). Clearly, there are many possible interventions available, and prioritization of those interventions for implementation among children has become one of the most important health policy goals for the governments, especially in low and middle income countries (8). They are calling for better evidence on the efficacy, effectiveness and cost-effectiveness of each intervention, and also better information on the patterns of child mortality burden in their countries (8). Therefore, understanding the causes of child mortality and the effectiveness of the preventive and treatment interventions has become one of the main interests of the global health community. The ‘Child Survival Series’ published by The Lancet is the earliest and one of the best examples (5,9-12).

The controversy over the true value of many child health interventions still rekindle debates now and then. First, once a few published randomized controlled trials show effectiveness of an intervention, it becomes unethical to scale up the evaluation only to deprive the control arm of apparent benefits. Second, the diversity of study settings and contexts produces enormous confounding and distorts generalization. Third, due to resource constraints, not every study can achieve sufficiently large simple size. Many of them eventually become underpowered, which leads to diverse and often conflicting results. Furthermore, mortality is rarely – if ever – an acceptable outcome of an intervention trial, and the introduction of proxy indicators creates uncertainty over the true effects on mortality and causes ambiguous conclusions. The most recent effort to integrate estimated effectiveness of interventions on child mortality has been published in 2 supplements of the *International Journal of Epidemiology* and *BMC Public Health*, providing the theoretical and methodological background information for the ‘Lives Saved Tool (LiST)’ (13). LiST is an evidence-based software module that allows prediction of the impact of scaling up different child health interventions at the national, regional and global level. Although this project is designed and performed to produce reasonably conservative estimates, the primary information gap is still an open issue and that new evidence from any proper sources would further refine the estimates.

One of the potential sources of this kind is academic literature in the Chinese language. The research production of Chinese academics for the past few decades, which is being published in more than nine thousands of Chinese academic periodicals, has recently been digitalized and made available in the public domain. It has been proven that strategically retrieving and analysing qualified publications in the Chinese literature contributes significant evidence and updates to the understanding and knowledge on various topics in the field of child health epidemiology (14). The aim of this study was to systematically identify and assess the evidence from Chinese literature sources on the efficacy and effectiveness of child health interventions in China.

## METHODS

The China National Knowledge Infrastructure (CNKI) databases were searched for the period January 1980 to March 2011. CNKI is the most complete source of academic information in China and represents the Chinese equivalent of PubMed or the Web of Knowledge (15). A systematic search of CNKI was performed to identify clinical and community-based studies with primary data reporting either efficacy or effectiveness of child health interventions relevant to neonates, infants, pre-school children or parents in China. Studies were included if: the total cohort was more than 50 subjects; the study design was prospective; the sample population was exclusively Chinese who lived in China at long-term residency base (16,17).

The search terms used were various combinations from a set of terms for ‘efficacy’ and ‘effectiveness’ in their Chinese equivalences, ie, ‘效果’, ‘评价’, ‘影响’, etc. and a set of terms for each intervention in their Chinese equivalences as well, eg, ‘叶酸’, ‘维生素B’, ‘补充’, etc. for ‘folic acid (vitamin B) supplementation’. The first step was to search prevention and treatment interventions targeting causes of child death in general (early development- related excluded). As this search strategy aimed to be as inclusive as possible, it resulted in 6476 publications which mentioned 32 interventions (**Table 1**). The first level screening based on titles left 649 studies for further identification as the majority were not relevant at all to the topic of ‘efficacy’ or ‘effectiveness’. The abstract screening then left 136 relevant records for full text retrieving. Eventually, 59 studies (up to 1% of the initial screen) for 20 interventions were retained (**Table 2**). The most common reasons for exclusion were: 1. the total sample cohort was less than 50 subjects, considering that the small sample size might lead to large errors; 2. study design was retrospective, which might cause massive recall bias; 3. the study method was not sufficiently described to understand or interpret the results.

**Table 1:** List of 32 interventions chosen for the review*

Preventive interventions
• Antenatal steroids to prevent preterm birth
• Antibiotics for premature rupture of membranes
• Breastfeeding (exclusively for 6 mo)
• Calcium supplementation to prevent pre-eclampsia and eclampsia
• Clean delivery practices
• Complementary feeding
• Detection and management of breech births (Caesarean section)
• Folic acid (vitamin B) supplementation
• Hib vaccination to prevent pneumonia
• Insecticide-treated materials
• Intermittent presumptive treatment for malaria in pregnancy
• Kangaroo mother care
• Labour surveillance (including partograph) for early diagnosis of complications
• Measles vaccination
• Nevirapine and replacement feeding (where possible) to prevent HIV transmission
• Newborn temperature management
• Prevention and management of hypothermia
• Syphilis detection and treatment
• Tetanus toxoid (neonatal)
• Vitamin A supplementation
• Water, sanitation, hygiene
• Zinc supplementation

Treatment interventions
• Antibiotics for dysentery
• Antibiotics for neonatal sepsis
• Anti-malarials
• Community-based pneumonia case management, including antibiotics
• Corticosteroids for preterm labour
• Detection and treatment of asymptomatic bacteriuria
• Newborn resuscitation
• Oral rehydration therapy
• Vitamin A
• Zinc for treatment of diarrhoea

*The interventions were chosen based on *Lancet*’s Child Survival Series list of interventions (5) and supplemented with any additional interventions on which at least 1 publication in Chinese literature could be found during the period 1980–2011.

**Table 2:** The list of 59 retained studies for 20 interventions

Preventive interventions	Key search terms	General search terms	Crude hits	Title hits	Abstract hits	Final hits
Antibiotics for premature rupture of membranes	抗生素;胎膜早破	效果;评价;影响;作用; 干预	309	26	2	1
Clean delivery practices	科学接生	效果;评价;影响;作用; 干预	30	16	4	1
Complementary feeding	辅食添加; 辅食喂养	效果;评价;影响;作用; 干预	147	23	13	7
Folic acid (vitamin B) supplementation	叶酸;维生素B;补充	效果;评价;影响;作用; 干预	123	21	12	2
HiB vaccination to prevent pneumonia	Hib疫苗	效果;评价;影响;作用; 干预	124	34	2	1
Kangaroo mother care	袋鼠式护理;皮肤接触护理	效果;评价;影响;作用; 干预	60	31	15	7
Labour surveillance (including partograph) for early diagnosis of complications	产前检查; 并发症;早期诊断	效果;评价;影响;作用; 干预	366	27	6	2
Measles vaccination	麻疹疫苗	效果;评价;影响;作用; 干预	68	21	5	2
Nevirapine and replacement feeding (where possible) to prevent HIV transmission	奈韦拉平;替代喂?; 艾滋病	效果;评价;影响;作用; 干预	156	44	2	1
Newborn temperature management	新生儿; 温度管理	效果;评价;影响;作用; 干预	1	1	1	1
Syphilis detection and treatment	梅毒	效果;评价;影响;作用; 干预	184	12	10	4
Tetanus toxoid (neonatal)	破伤风;类毒素;新生儿	效果;评价;影响;作用; 干预	197	9	3	2
Vitamin A supplementation	维生素A;维他命A	效果;评价;影响;作用; 干预	558	56	10	2
Water, sanitation, hygiene	水与环境卫生(WES)项目	效果;评价;影响;作用; 干预	19	8	2	1

Treatment interventions						
Antibiotics for dysentery	痢疾;抗生素	效果;评价;影响;作用; 干预	263	5	1	1
Corticosteroids for preterm labour	糖皮质激素; 早产	效果;评价;影响;作用; 干预	231	12	6	1
Newborn resuscitation	新生儿复苏	效果;评价;影响;作用; 干预	969	29	18	11
Oral rehydration therapy	口服补液疗法	效果;评价;影响;作用; 干预	112	15	1	1
Vitamin A	维生素A;维他命A	效果;评价;影响;作用; 干预	138	28	2	2
Zinc for treatment of diarrhoea	锌;腹泻;治疗	效果;评价;影响;作用; 干预	170	39	12	11

## RESULTS

**Figure 1:**
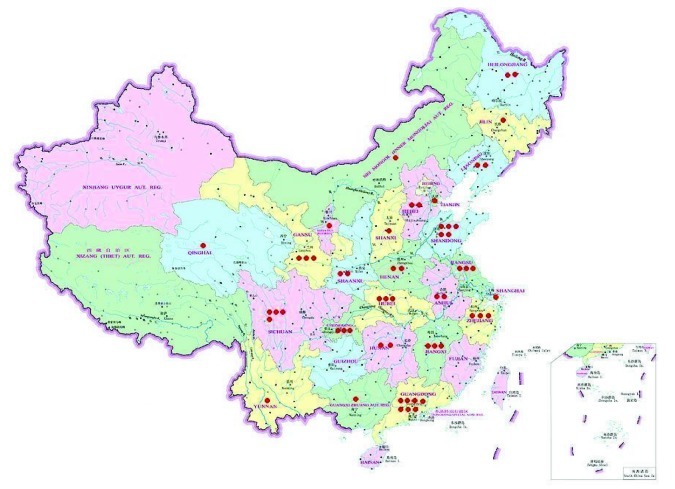
Geographical distribution of 59 retained studies during 1980–2011.

**Figure 2:**
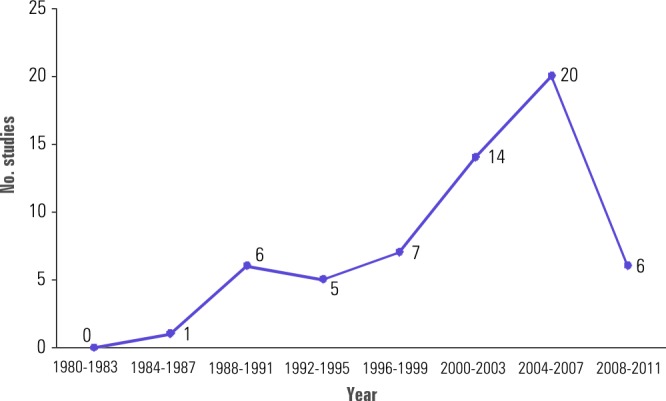
The time trend of 59 retained studies during 1980–2011.

The distribution of the 59 included studies clustered mainly in the eastern and central regions of China (**Figure 1**). The latest classification of Chinese regions based on regional macro-economy development recommended dividing China into the eastern, central and western regions. Of the three macro-regions, Western China suffered the most from economical disadvantages. This also provides insight into a further discrepancy: the retained studies conducted in Eastern and Central China were generally led by major university-affiliated hospitals, which were in control of the key resources, such as the provincially leading research and teaching hubs and/or networks. In Western China, however, the retained studies were mostly conducted through cooperation with international organizations, as the overseas aid programmes. Also, an analysis of the period 1980 to 2011, which separated the included studies into 4-year time-periods by frequency, showed the time trend of a steady increase until the period 2004–2007. This was followed by a sharp drop during the period 2008–2011, to the level of activity observed back in 1992–1995 (**Figure 2**).

All the included studies that evaluated a total of 20 interventions were focused on addressing efficacy or effectiveness as the primary outcome. Among preventive interventions (14 interventions), most studies were dedicated to complementary feeding (7 studies), kangaroo mother care (7 studies) and syphilis detection and treatment (4 studies) (**Table 3**). Among treatment interventions (6 interventions), the most frequently studied were zinc for treatment of diarrhoea (11 studies) and newborn resuscitation (9 studies) (**Table 4**). Within each intervention, the results from individual studies generally supported each other, providing evidence on the positive effects for all interventions. However, several peculiarities need to be noted: for complementary feeding, each study adopted different food formulas and this may be due to the widespread geographic distribution. For Kangaroo mother care, the research interests varied dramatically, which made the targeting outcomes very diverse – including body temperature management, pain management, weigh gain, physiological and behavioural factors, etc. For syphilis detection and treatment, it was notable that one community-based study conducted in Guangdong province and recruiting 186 517 subjects reported an impressive 61.90% reduction rate during a period of two years. For zinc for treatment of diarrhoea, although OER and anti-diarrhoea duration were common indicators, the ingredients and portions of zinc formula adopted in each study were not consistent. Finally, for newborn resuscitation the universal outcome indicators employed in each study were Apgar-5 and Apgar-10, while Apgar-1 and Apgar-7 occasionally reported with other specific outcomes such as mortality, morbidity of asphyxia, pneumonia and infections.

**Table 3:** Three most investigated preventive child health interventions in Chinese literature

Study No	Year	Province	Cohort site	Cohort No.	Sample size (Intervention1)	Sample size (Intervention2)	Sample size (Controls)	Outcome	Indicators of the outcome				
									1		2	3	4
• Kangaroo mother care													
1	2009	Zhejiang	Hospital	–	100	–	100	Body temperature management	Temperature recovery speed (hr): I = 1.665 ± 1.36, C = 6.635 ± 3.838, *P* < 0.05	–		–	–
2	2008–2009	Guangdong	Hospital	–	50	–	50	Venepuncture pain management	Heart rates (/min): I<C, (8-14), *P* < 0.05	Blood oxygen rate (%): I<C, (1-5), *P *< 0.05		–	–
3	2007	Hebei	Hospital	–	80	–	80	Body temperature management	Temperature recovery speed: I = 38(<4h), C = 18(<4h), *P* < 0.05	–		–	–
4	2007–2008	Guangdong	Hospital	–	30	–	30	Weight gain	Weight gain in the 28th day: I = 835g, C = 750g, *P* < 0.0005	–		–	–
5	2006–2008	Hunan	Hospital	–	60	–	60	Physiological and behaviour indicators	Heart rates (/min): I = 142.4 ± 19.1, C = 157.1 ± 24.8, *P* < 0.01	Blood oxygen rate (%): I = 91.7 ± 4.6, C = 87.9 ± 9.1, *P* < 0.01		–	–
6	2004–2009	Shanxi	Hospital	–	55	–	53	Pain management	Heart rates (/min): I = 152.77 ± 15.98, C = 162.34 ± 22.02, *P* < 0.05	Blood oxygen rate (%): I = 94.03 ± 10.89, C = 93.08 ± 10.62, *P* < 0.05		–	–
7	1999–2002	Guangdong	Hospital	–	54	–	50	Prognosis of very LBW	MDI: I = 99 ± 12, C = 89 ± 10, *P* < 0.01	PDI:, I = 91 ± 10 C = 83 ± 11, *P* < 0.05		–	–

• Complementary feeding												
1	2007–2008	Guangxi	Community	–	100	–	100	–	Percentage-Nutrition (P0-P100): I>C, *P* < 0.05	–	–	–
2	2006–2008	Jiangsu	Hospital	–	206	–	175	–	Hb (g/L): I = 80.74 ± 13.24 C = 66.43 ± 12.57, *P* < 0.05	IDA: I = 9(4.5%) C = 33(19.4), *P* < 0.05	–	–
3	2004	Gansu	Community	–	1000	500	127	–	DQ: I>C, *P* < 0.0001	–	–	–
4	2003	Guangdong	Hospital	–	251	–	237	–	Malnutrition: I = 9, C = 42, *P* < 0.01	–	–	–
5	2002	Jiangsu	Hospital	–	52	–	52	–	Anaemia(%): I = 17.31, C = 38.46, *P* < 0.05	–	–	–
6	1997	Liaoning	Hospital	–	107	–	34	–	Standard BW: I = 64(87.7), C = 21(61.8), *P* < 0.01	–	–	–
7	1994	Sichuan	Hospital	–	45	60	58	–	BW, Height, HC: I>C, *P* < 0.05	–	–	–

• Syphilis detection and treatment												
1	2006–2009	Inner Mongolia	Hospital	–	168	–	32	–	Syphilis(+): 72 children born to Interventions; 19 children born to Controls	–	–	–
2	2005	Guangdong	Community	159017	–	–	–	–	125(syphilis child cases in theory for 2005) – 4 (syphilis child cases in 2005)	–	–	–
3	2001–2005	Guangdong	Hospital	–	50	–	13	–	Syphilis(+): 8 children born to Interventions; 10 children born to Controls	–	–	–
4	2001–2002	Guangdong	Community	186517	–	–	–	–	Reduction: 61.90%	–	–	–

I – interventions, C – controls, LBW – low birth weight, MDI – mental developmental index, PDI – psychomotor developmental index, Hb – hemoglobin concentration, IDA – iron deficiency anaemia, DQ – developmental quotient, BW – birth weight, HC – head circumference

**Table 4:** Two most investigated treatment child health interventions in Chinese literature

Study No	Year	Province		Cohort site	Cohort No.	Sample size (Intervention1)	Sample size (Intervention2)	Sample size (Controls)	Outcome	Indicators of the outcome			
										1	2	3	4
• Zinc for treatment of diarrhoea													
1	2009	Shandong		Hospital	–	65	–	60	–	OER (%): I = 96.92, C = 78.33, *P* < 0.05	Anti-diarrhoea duration (days): I = 6 ± 1.05, C = 7 ± 1.02, *P* < 0.05	–	–
2	2008–2009	Jiangxi		Hospital	–	90	–	90	–	OER (%): I = 90.0, C = 60.0, *P* < 0.05	Anti-diarrhoea duration (days): I = 3.11 ± 2.41, C = 5.32 ± 2.11, *P* < 0.01	–	–
3	2008–2009	Tianjing		Hospital	–	45	–	42	–	OER (%): I = 86.3, C = 76.2, *P* < 0.05	–	–	–
4	2008–2009	Liaoning		Hospital	–	55	–	55	–	OER (%): I = 89.09, C = 65.45, *P* < 0.01	–	–	–
5	2007–2009	Shaanxi		Hospital	–	40	–	40	–	OER (%): I = 92.5, C = 80.0, *P* < 0.05	Anti-diarrhoea duration (days): I = 2.81 ± 0.83, C = 4.21 ± 1.98, *P* < 0.01	–	–
6	2007–2009	Sichuan		Hospital	–	120	–	120	–	OER (%): I = 93.4, C = 82.5, *P* < 0.05	–	–	–
7	2007–2009	Jiangxi		Hospital	–	60	–	60	–	OER (%): I = 93.4, C = 73.2, *P* < 0.01	Anti-diarrhoea duration (hrs): I = 48.92 ± 3.02, C = 100.23 ± 3.16, *P* < 0.05	–	–
8	2007	Hubei		Hospital	–	55	–	50	–	Anti-diarrhoea duration (hrs): I = 43 ± 6, C = 49 ± 5, *P* < 0.01	–	–	–
9	2006–2008	Chongqing Hospital			–	164	–	168	–	OER (%): I = 92.07, C = 74.41, *P* < 0.01	Anti-diarrhoea duration (days): I = 3.11 ± 1.41, C = 4.07 ± 2.12, *P* < 0.01	–	–
10	2005–2007	Sichuan	Hospital		–	95	–	91	–	OER (%): I>CP; <0.01	–	–	–
11	2005–2006	Shandong	Hospital		–	124	–	126	–	OER (%): I = 98, C = 92, *P* < 0.05	–	–	–

• Newborn resuscitation												
1	2003–2006	Shandong	Hospital	–	3050	–	2850	–	Asphyxia (%): I = 0.4, C = 2.1, *P* < 0.01	–	–	–
2	2002–2005	Qinghai	Hospital	–	682	–	1318	–	Mortality (%): I = 15, C = 38, *P* < 0.05	–	–	–
3	2001–2006	Hubei	Hospital	–	13905	–	7808	–	Asphyxia (%): I = 0.48, C = 2.7, *P* < 0.01	–	–	–
4	2000–2003	Henan	Hospital	–	168	–	55	–	Apgar-5: I = 8.0 ± 1.0, C = 6.3 ± 0.8, *P* < 0.01	Apgar-10: I = 9.0 ± 1.1, C = 8.0 ± 0.5, *P* < 0.01	–	–
5	1999	Anhui	Hospital	–	143	–	143	–	Apgar-7: I = 3.4, C = 12.5, *P* < 0.05	Pneumonia (%): I = 1.3, C = 6.9, *P* < 0.05	Infections (%): I = 1.3, C = 6.2, *P* < 0.05	–
6	1997–2000	Sichuan	Hospital	–	52	–	56	–	Pneumonia: I = 9, C = 2, *P* < 0.05	HIE: I = 9, C = 1, *P* < 0.05	Apgar-5: I = 46, C = 50, *P* < 0.05	Apgar-10: I = 50,C = 52 *P* < 0.05
7	1997–1998	Jilin	Hospital	–	2478	–	782	–	Asphyxia (%): I = 5.29, C = 15.22, *P* < 0.01	–	–	–
8	1993–1997	Shandong	Hospital	–	54	–	63	–	Survival rate (%): I = 100, C = 90.47, *P* < 0.005	–	–	–
9	1989, 1997	Shaanxi	Hospital	–	1349	–	2414	–	Asphyxia (%): I = 2.3, C = 4.4, *P* < 0.05	Apgar-1: I = 6.1, C = 3.7, *P* < 0.01	Apgar-5: I = 9.7, C = 7.7, *P* < 0.01	–

I – interventions, C – controls, OER – overall effect rate, HIE – hypoxic ischemic encephalopathy

The evidence on efficacy or effectiveness of the 32 interventions conducted in Chinese children in the Chinese literature was either of comparable quality, or more informative than the available reports on China in the English literature (**Table 5**). Although the selected English literature sources showed a substantial and strikingly increasing number of publications on child health interventions during the same period, studies on intervention effectiveness exclusively in Chinese children were very rarely published, and the few rare studies were unlikely to scale-up a systematic analysis or meta-analysis for any of the 32 selected interventions in this paper.

**Table 5:** The remaining 15 child health interventions with addressed effectiveness in Chinese literature

PREVENTIVE INTERVENTIONS													
Study No.	Year	Province	Cohort	Cohort Size	Sample size (Intervention 1)	Sample size (Intervention 2)	Sample size (Intervention 3)	Sample size (Control)	Outcome	Indicators of outcome			
										1	2	3	4
• Folic acid (vitamin B) supplementation													
1	1996-1997	Zhejiang	Hospital	–	2265	–	–	2265	Congenital heart diseases	RR = 1.77(95%CI: 1.082–2.466); AR = 1.29% ARP = 43.63% *P* < 0.01	–	–	–
2	1993–1995	National	Community	–	130142	–	–	117689	NTD	120(NTDs)/130142 137(NTDs)/117689 *P* < 0.01	–	–	–

• Tetanus toxoid (neonatal)													
1	1991–1993	Hunan	Community	–	170	–	–	170(self)	–	IU/ml(mothers) increased = 0.137–0.008 IU/ml(children) = 0.054 *P* < 0.01	–	–	–
2	1986–1987	Ningxia	Community	–	67	–	–	41	–	IU/ml >0.01: 89.55% of children born to Interventions; 14.63% of children born to Controls *P* < 0.01	–	–	–

• Antibiotics for premature rupture of membranes													
1	1992–1997	Henan	Hospital	–	30	–	–	30	–	BW: I = 2580g; C = 2391g *P* < 0.01	1 min Apgar ≤7: I = 4; C = 9 *P* < 0.01	Infections: I = 5; C = 7, *P* < 0.01 Intracranial haemorrhage: I = 5; C = 5 *P* < 0.01	Death: I = 0; C = 2 *P* < 0.01

• Labour surveillance (including partograph) for early diagnosis of complications													
1	2006–2008	Zhejiang	Hospital	–	11186	–	–	1066	–	RDS: I = 290; C = 52 *P* < 0.01	LBW: I = 551; C = 88 *P* < 0.01	Preterm birth:I = 554; C = 95 *P* < 0.01	Caesarean section rate: I = 68; C = 113 *P* < 0.01
2	2006–2007	Shandong	Hospital	–	226	–	–	208	–				

• Clean delivery practices													
1	1991–1993	Heilongjiang	Hospital	–	355	–	–	366	–	Death (Children): I = 9; C = 5 *P* < 0.01	–	–	–

• Newborn temperature management													
1	2002–2003	Gansu	Hospital	–	715	–	–	1096	–	neonatal sclerodema: I = 3 C = 10 *P* < 0.01	–	–	–

• Hib vaccination to prevent pneumonia													
1	2006	Shanghai	Community	–	372	-	-	305	–	Hib+ (%): I = 9.8; C = 3.8 *P* < 0.01	–	–	–

• Water, sanitation, hygiene													
1	1996–2000	Gansu	Community	21 villages	–	–	–	–	–	Sanitation per household, population educated, children hygiene behaviour positive change rate are all increased	–	–	–

• Vitamin A supplementation													
1	2001–2005	Chongqing	Institute	–	64	–	–	61	–	Hb (g/L): I = 122.0 ± 7.5; C = 120.3 ± 7.87 *P* < 0.001	–	–	–
2	1991–1992	Hebei	Community	–	327	–	–	343	–	Z value:I>C *P* < 0.01	–	–	–

• Nevirapine and replacement feeding (where possible) to prevent HIV transmission													
1	2005	Yunnan	Community	–	61	61	-	-	-	Reduction: 7.0%	–	–	–

• Measles vaccination													
1	2000–2007	Guizhou	Community	25302	–	–	–	–	–	Morbidity: 26.68/100000(2002) 3.52/100000(2003) 0.20/100000(2007)	–	–	–
2	1991	Hubei	Community	503	–	–	–	–	–	Reduction in 6 y: 59.22%	–	–	–

TREATMENT INTERVENTIONS													
Study No.	Year	Province	Cohort	Cohort Size	Sample size (Intervention 1)	Sample size (Intervention 2)	Sample size (Intervention 3)	Sample size (Control)	Outcome	Indicators of outcome			
										1	2	3	4
• Corticosteroids for preterm labour													
1	2000	Anhui	Hospital	–	–	18	19	–	5	Hyaline membrane disease (<34 weeks gestation): I<C *P* < 0.001	–	–	–

• Oral rehydration therapy													
1	2001–2005	Heilongjiang	Hospital	–	60	–	–	60	–	Temperature recovery speed (days): I = 1.51 ± 0.89;C = 3.87 ± 2.03 *P* < 0.01	Diarrhoea recovery speed (days): I = 2.97 ± 1.45;C = 5.72 ± 2.43 *P* < 0.01	General recovery speed (days): I = 4.70 ± 1.98;C = 7.28 ± 2.86 *P* < 0.01	–

• Antibiotics for dysentery													
1	1994–1996	Jiangsu	Hospital	–	24	30	23	–	–	2 preferred based on temperature recovery speed and anti–diarrhoea duration: Both Ps <0.01	–	–	–

• Vitamin A													
1	2003–2006	Jiangxi	Hospital	–	42	–	–	35	Recurrent respiratory infections	Temperature recovery speed (days): I = 3.5; C = 6.0 *P* < 0.01	Diarrhoea recovery duration (days): I = 5; C = 8 *P* < 0.01	Diarrhoea recovery duration (days): I = 5; C = 8 *P* < 0.01	Recurrence (%): Interventions<Controls *P* < 0.05
2	1990–2000	Chongqing	Hospital	–	30	–	–	30	Measles	VA (µmol/L): I = 1.17 ± 0.12; C = 0.84 ± 0.24 *P* < 0.01	Cough recovery duration (days): I = 8.12 ± 1.24: C = 9.36 ± 1.68 *P* < 0.01	Diarrhoea recovery duration (days): I = 7.88 ± 1.87; C = 9.13 ± 1.20 *P* < 0.01	Complications (%) Interventions<Controls Ps <0.05

I – intervention, C – control, RR – risk ratio, AR – attribute ratio, ARP – attribute ratio percentage, RDS – respiratory distress syndrome, Hb – hemoglobin concentration

## DISCUSSION

This paper represents the very first attempt to systematically investigate the accessibility, quantity and quality of the research production of Chinese academics over the period of the past 30 years on efficacy and effectiveness of child health interventions in China. It is possible that a parallel review and/or the extended systematic review of other digital Chinese-language databases such as Chongqing VIP and Wanfang would have identified slightly more studies. However, the initial searching within the two additional databases did not seem to add any further evidence (my search on February 15, 2011), and this might due to the extremely massive overlap in indexed journals of the three domains (15,18,19). It is very unlikely that substantial omissions could have drawn dramatically different conclusions on quantity assessment from those that this paper offers.

Conducting studies which evaluate interventions generally not only requires substantial financial support and high-level technical expertise, but it is also subject to various vital factors, such as ethical approval, unique local conditions/customs and liaisons with multiple organizations, to name a few. Usually, a single team of researchers has to invest tremendous efforts to ensure smooth workflow for years to achieve the ultimate outcome, either positive or negative, even possessing sufficient essential resources. This may explain why, in the global context, the research production on intervention evaluation is relatively scarce and the relevant studies with large sample sizes and robust study designs are extremely valuable.

According to GRADE criteria, the retained studies in the Chinese literature are generally of modest quality (17). Relatively small sample sizes and wide confidence intervals make it unjustified that all retained studies should be expected to show positive effects of the investigated interventions, implying a likely publication bias. This has not ridden the Chinese literature only, as there is a traditional academic and industrial resistance to reporting and pursuing publication of negative effects, especially where the previous evaluation showed benefits. China may consider incorporating the existing national ethics approval system with a mandatory national trail registration system in order to keep all the trails conducted in China well tracked and ensure that the final outcomes are reported and made public.

Despite insufficient information to fully assess the current situation of child health interventions in depth in China, the urban-rural discrepancy in study distribution and funding resources is particularly notable. While the studies in Eastern and Central China were conducted by regional leading institutes as scientific research projects, Western China, the most remote and mountainous region of the country, seems to still be reliant on overseas aid to implement child health interventions. The longstanding urban-rural socio-economical imbalance results in limited academic and industrial activities focusing on the most underdeveloped region of China, where implementing, evaluating and further developing appropriately customized interventions may achieve highest potential impact on child mortality reduction and other aspects of child health in China as a whole.

Comprehensive and accurate data and evidence on child health interventions are indispensable for prioritizing prevention and treatment strategies in order to achieve optimal policy decisions. It is clear that considerable research interests and initiatives from both inside and outside the country have been concentrating on child health interventions, especially preventive interventions in China. How to best harness and facilitate those potential strengths in this still new and fast growing field could be the next opportunity and challenge that the country has to take.
